# HCV treatment rates and sustained viral response among people who inject drugs in seven UK sites: real world results and modelling of treatment impact

**DOI:** 10.1111/jvh.12338

**Published:** 2014-10-07

**Authors:** N K Martin, G R Foster, J Vilar, S Ryder, M E Cramp, F Gordon, J F Dillon, N Craine, H Busse, A Clements, S J Hutchinson, A Ustianowski, M Ramsay, D J Goldberg, W Irving, V Hope, D De Angelis, M Lyons, P Vickerman, M Hickman

**Affiliations:** 1 School of Social & Community Medicine, University of BristolBristol, UK; 2 Social and Mathematical Epidemiology Group, London School of Hygiene and Tropical MedicineLondon, UK; 3 Blizard Institute, Queen Mary's University of LondonLondon, UK; 4 Pennine Acute Hospitals NHS TrustGreater Manchester, UK; 5 Nottingham University Hospitals NHS TrustNottingham, UK; 6 Plymouth Hospital NHS TrustPlymouth, UK; 7 University of Bristol Health TrustBristol, UK; 8 University of DundeeDundee, UK; 9 Health Protection WalesBangor, Wales, UK; 10 Glasgow Caledonian UniversityGlasgow, UK; 11 Health Protection ScotlandGlasgow, UK; 12 Public Health EnglandLondon, UK; 13 University of NottinghamNottingham, UK; 14 MRC Biostatistics UnitCambridge, UK

**Keywords:** antiviral treatment, direct acting antivirals, hepatitis C virus, injecting drug users, people who inject drugs, prevention, sustained viral response

## Abstract

Hepatitis C virus (HCV) antiviral treatment for people who inject drugs (PWID) could prevent onwards transmission and reduce chronic prevalence. We assessed current PWID treatment rates in seven UK settings and projected the potential impact of current and scaled-up treatment on HCV chronic prevalence. Data on number of PWID treated and sustained viral response rates (SVR) were collected from seven UK settings: Bristol (37–48% HCV chronic prevalence among PWID), East London (37–48%), Manchester (48–56%), Nottingham (37–44%), Plymouth (30–37%), Dundee (20–27%) and North Wales (27–33%). A model of HCV transmission among PWID projected the 10-year impact of (i) current treatment rates and SVR (ii) scale-up with interferon-free direct acting antivirals (IFN-free DAAs) with 90% SVR. Treatment rates varied from <5 to over 25 per 1000 PWID. Pooled intention-to-treat SVR for PWID were 45% genotypes 1/4 [95%CI 33–57%] and 61% genotypes 2/3 [95%CI 47–76%]. Projections of chronic HCV prevalence among PWID after 10 years of current levels of treatment overlapped substantially with current HCV prevalence estimates. Scaling-up treatment to 26/1000 PWID annually (achieved already in two sites) with IFN-free DAAs could achieve an observable absolute reduction in HCV chronic prevalence of at least 15% among PWID in all sites and greater than a halving in chronic HCV in Plymouth, Dundee and North Wales within a decade. Current treatment rates among PWID are unlikely to achieve observable reductions in HCV chronic prevalence over the next 10 years. Achievable scale-up, however, could lead to substantial reductions in HCV chronic prevalence.

## Introduction

Chronic hepatitis C virus (HCV) infection is a leading cause of liver disease, death and disability [1,2]. In the United States, more people die each year from HCV than HIV [3]. Preventing HCV transmission is critical for averting future liver disease [4]. In many developed countries and developing countries with injecting drug use, this principally involves controlling HCV infection among people who inject drugs (PWID) [5,6]. For example, in the United Kingdom, nearly 200 000 people (0.5% of adults aged 15–60) are infected with HCV, of which over 85% acquired HCV through injecting drug use [7].

The combination of opiate substitution treatment (OST) and high-coverage needle and syringe programmes (NSP) can reduce HCV transmission among PWID [8,9]. However, it is unlikely that OST and high-coverage NSP can be sustained sufficiently to achieve substantial reductions in HCV prevalence among PWID [10], and no community has achieved a marked reduction with this approach. Model projections have suggested that HCV treatment among PWID could be effective as primary prevention (i.e. ‘treatment as prevention’) [11–16] and that treating PWID may be more cost-effective than treating ex/former PWID with no ongoing transmission/infection risk in many settings [12]. Indeed, mathematical models have suggested that scaling up of HCV treatment is required to achieve a reduction in HCV prevalence of over 40% among PWID in the next decade [17].

Epidemiological data suggest that PWID can be treated and achieve similar sustained viral response (SVR) as other groups, especially if incorporated with OST [18,19]. Nonetheless, overall HCV treatment rates remain low, and evidence on the number of PWID treated is sparse, as data on injecting status are not always routinely collected during HCV treatment [20]. The imminent availability of interferon-free direct acting antiviral (IFN-free DAA) therapies will dramatically change the HCV treatment landscape – if once-daily all-oral interferon-free regimes with high SVR rates (∽90%), short duration (12–24 weeks), low toxicity, high barrier to resistance and reduced monitoring can be achieved and translated to real world settings [21–28].

In this study, we determined current treatment rates and outcomes among PWID in selected sites in the United Kingdom and projected the likely impact of these treatment rates on chronic HCV prevalence among PWID over the next decade, as well as considered the impact of HCV treatment scale-up and use of new IFN-free DAAs.

## Methods

### Service evaluation

The United Kingdom, like many other countries, lacks routine data on the number of people who inject drugs (PWID) treated for HCV from public health or clinical reporting systems [29]. Therefore, we undertook an anonymous service evaluation of seven UK sites: five in England [Bristol, East London (Newham, Hackney and Tower Hamlets), Plymouth, Nottingham, and Manchester], one in Scotland (Dundee and Tayside, including Angus, Perth and Kinross) and one in Wales comprising North West and North East Wales (Betsi Cadwaladr). Dependent heroin use is a chronic relapsing condition – with no absolute definition of long-term injecting cessation [30,31]. We classified recent PWID as people who at the time of their HCV treatment were currently injecting, reported injecting in the last 3 years and/or were on OST. One additional potential site was excluded because consistent data on number of PWID treated could not be collected. Lead physicians and nurses (in hepatology or infectious diseases) in each site provided information on the number of PWID treated during 2009–2011 and HCV treatment SVR rates by genotype with pegylated interferon and ribavirin (pegIFN/RBV). SVR data for recent PWID were pooled to generate estimates by genotype for use in model projections (Table1). There was moderate to high heterogeneity between sites; therefore, we used a random effects model including the site as a random effect for the estimates of SVR – with each site contributing approximately equally to the weighted average. We estimated two sets of SVR – either excluding cases with missing follow-up information (a per protocol analysis that assumes information on SVR was missing completely at random, designated ‘per protocol SVR’ and a best case scenario) or classifying people with missing SVR as treatment failures (designated as ‘Intention To Treat’ ITT SVR and a worst case scenario).

**Table 1:** Service evaluation results

Parameter	Value/Range	Notes
Sustained viral response rate		
Peg-IFN/RBV G1/4 ITT*	45% [95%CI 33–57%]	Sampled from a uniform distribution in model projections
Peg-IFN/RBV G2/3 ITT*	61% [95%CI 46–76%]	
Peg-IFN/RBV G1/4 per protocol**	59% [95%CI 46–71%]	
Peg-IFN/RBV G2/3 per protocol**	82% [95%CI 69–94%]	
Number PWID treated per year		
Bristol	18	
East London	25	
Manchester	63	
Nottingham	32	
Plymouth	17	
Tayside/Dundee	34	
North Wales	18	
Treatment rate per 1000 PWID in 2013		
Bristol	4.1–5.6	Calculated from estimated number PWID (see appendix); uncertainty due to uncertainty in number of PWID. Sampled from a uniform distribution in model projections
East London	4.2–10.4	
Manchester	15.8–27.4	
Nottingham	12.8–24.6	
Plymouth	8.5–15.5	
Tayside/Dundee	11.3–17.0	
North Wales	5.3–10.6	

PWID = people who use drugs; G1/4 = genotypes 1 and 4; G2/3 = genotypes 2 and 3; ITT: intention to treat.

* Analysis classifying people with missing SVR as treatment failures.

** Analysis excluding cases with missing follow-up information/missing classified as completely at random.

Additionally, we collated baseline data on HCV seroprevalence among PWID and number of PWID (appendix Tables S1,S2). Estimates for the number of PWID were used to calculate the annual HCV treatment rates per 1000 PWID for model projections (Table1, details in appendix), where the denominator is the total number of PWID (not the estimated number with chronic HCV).

### Mathematical model

We used a previously published dynamic, deterministic, compartmental model of HCV transmission and treatment among PWID [11]. The model included compartments for uninfected PWID (*X(t)*), PWID chronically infected with HCV (*C(t)*), PWID on antiviral treatment (*T(t)*) and PWID who failed antiviral treatment (F*(t)*). We tracked changes in the populations over time, *t*. As the model is dynamic, the risk of infection or reinfection for a PWID is proportional to HCV chronic prevalence, which changes over time. We did not assume any risk difference after treatment; reinfection risk is equal to primary infection risk. For details model and equations, see appendix.

#### Scenarios examined

We evaluated four treatment scenarios with varying SVR rates:

- SVR with pegIFN/RBV based on the intention-to treat-analysis (‘ITT SVR’) for 10 years with existing PWID treatment rates
- SVR with pegIFN/RBV ignoring patients missing SVR data post-treatment completion (‘per protocol SVR’) for 10 years with existing PWID treatment rates
- ITT SVR until 2015, with IFN-free DAAs for genotype 1 patients only (90% SVR [21–28]) from early 2016 onwards and a scale up of treatment to 26 per 1000 PWID (the upper estimate of what is currently achieved in Manchester and Nottingham) (‘conservative DAA scenario’)
- Per protocol SVR until 2015, with IFN-free DAAs for all genotypes (90% SVR [21–28]) from early 2016 onwards and a scale up of treatment to 26 per 1000 PWID (the upper estimate of what is currently achieved in Manchester and Nottingham) (‘optimistic DAA scenario’)

Clinical guidance in the United Kingdom recommended against treatment of PWID prior to 2002 [32], and PWID treatment rates prior to this study are unknown. Therefore, we assumed that current treatment rates have been in place for 5 years prior to 2014 (2009 onwards).

#### Multivariate uncertainty analyses

To consider the effect of uncertainty in the underlying parameters, we performed a multivariate probabilistic uncertainty analysis where 1000 parameter sets were randomly sampled from setting specific parameter distributions in Table1. For each of the 1000 parameter sets, the model was calibrated to the sampled HCV chronic prevalence in 2013 by varying the infection rate, *π*. The model was assumed to be in steady state prior to 2009, such that both the number of PWID and also the chronic HCV prevalence among PWID were stable. From 2009 to 2014, the model also took into account HCV treatment rates achieved by the sites and then was used to project the prevalence reductions in each setting for a further 10 years with current treatment rates or scaled-up treatment rates. For all projections, 95% intervals were generated from the multivariate uncertainty sampling. A linear regression analysis of covariance (ANCOVA) was performed on the 10-year relative prevalence reduction, and the proportion of the sum of squares contributed by each parameter was calculated to estimate the importance of individual parameters to the overall uncertainty [33]. All equations were solved using MATLAB, using the ordinary differential equation solver ODE45.

#### Parameters

A list of the parameters used in the modelling simulations can be found in appendix Tables S1,S2.

## Results

### Survey of treatment centres

We received data on a total of 1337 people treated for HCV in the participating sites; of which 927 were resident in the sites and 538 (58%) were classified as PWID (i.e. current or recent injectors or on OST), 54 (6%) recent injecting status was unknown, and 335 (36%) were exposed through other routes or last injected more than 3 years from date of HCV treatment and were not on OST.

**Figure 1:**
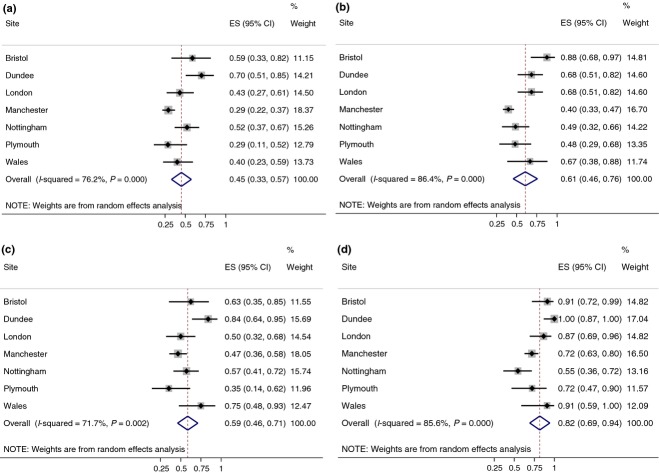
Pooled random effects model estimates of SVR (sustained viral response) by genotype and classification of missing data. (a) Genotype 1&4 ITT (missing follow-up classified as nonresponder/treatment failure) (b) Genotype 2&3 ITT (missing follow-up classified as nonresponder/treatment failure) (c) Genotype 1&4 per protocol (missing follow-up excluded/classified as completely at random) (d) Genotype 2&3 per protocol (missing follow-up excluded/classified as completely at random). ITT = intention to treat.

Annual HCV treatment rates varied from an estimated rate per 1000 PWID of <5 to over 25 (Table1). Individual and pooled estimates of SVR for PWID are presented in Fig.1, with pooled results listed in Table1. The pooled ITT SVR (assuming those lost to follow-up were treatment failures) was 61% (95%CI 46–76) for genotype 2/3 and 45% (95%CI 33–57) for genotype 1/4. The pooled per protocol SVR (ignoring missing follow-up data) was 82% (95%CI 69–94) for genotype 2/3 and 59% (95%CI 46–71) for genotype 1/4. There was weak evidence that SVR varied by site (Fig.1). We stratified sites by their treatments rates, defining settings as low treatment (<11 per 1000 PWID treatments: Bristol, East London, North Wales) and high treatment (≥11 per 1000 PWID treatments: Manchester, Nottingham, Plymouth, Dundee). High treatment sites tended to have lower SVR than low treatment sites: for genotype 2/3 ITT SVR, the odds ratio was 0.35 (95%CI 0.15–0.82) comparing sites with high versus low treatment rates adjusted for age, and for genotype 1/4 ITT, the odds ratio was 0.82 (95%CI 0.45–1.49) comparing high vs. low treatment sites after adjustment for age. None of the sites had systematic follow-up of successfully treated patients to assess reinfection.

### Model projections

**Figure 2:**
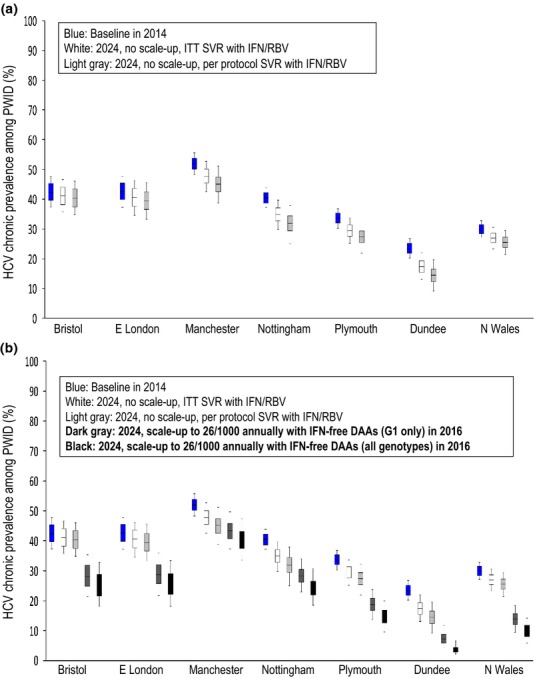
Model projections for HCV chronic prevalence among PWID in 2014 (blue) and in 2024 (white/gray/black) with various treatment scenarios. (a) Projections are shown for no treatment scale-up and using Peg-IFN/RBV ITT SVR rates from 2005 (white), no treatment scale-up and using Peg-IFN/RBV per protocol SVR rates from 2005 (light gray). (b) Projections are shown for no treatment scale-up and using Peg-IFN+RBV ITT SVR rates from 2005 (white), no treatment scale-up and using Peg-IFN+RBV per protocol SVR rates from 2005 (light gray), ‘conservative DAA scale-up scenario’ with ITT SVR until 2015, treatment scale-up to 26/1000 PWID and IFN-free DAAs for genotype 1 patients only in 2016 (dark gray), ‘optimistic DAA scale-up scenario’ with per protocol SVR until 2015, treatment scale-up to 26/1000 PWID and IFN-free DAAs for all genotypes in 2016 (black). Boxes show the interquartile range, with whiskers indicating the 95% intervals.

Figure2a shows the HCV chronic prevalence among PWID at 10 years using the ITT SVR or per protocol SVRs for peg-IFN+RBV assuming a continuation of current treatment rates. With current treatment rates and the ITT SVR, modest impact will be achieved at 10 years, with no settings achieving absolute chronic prevalence reductions of 10%. At 10 years, the 95% interval of projected HCV chronic prevalence among PWID would largely overlap with the current estimates (Fig.2a) including from 48–56% *vs* 43–53% in Manchester, 37–48% *vs* 36–47% in Bristol, 37–48% *vs* 35–46% in East London, 48–56% v*s* 30–40% in Nottingham, 30–37% *vs* 25–34% in Plymouth, 20–27% *vs* 23-31% in North Wales, 20–27% *vs* 13–22% in Dundee. Using the higher per protocol SVR for peg-IFN/RBV increases the potential impact (by approximately 30–40%), but the uncertainty estimates still overlap. Two settings potentially achieve absolute chronic prevalence reductions of 10%: Nottingham (4–11% absolute chronic prevalence reduction) and Dundee (6–11%).

Conversely, if current treatment rates are scaled up to 26 per 1000 PWID annually (the upper range of what is estimated to currently occur in Manchester and Nottingham) and IFN-free DAAs become available in 2016, then the model predicts substantial prevention impact (Fig.2b). For instance, if IFN-free DAAs only become available for genotype 1 patients (with genotype 2/3 remaining on pegIFN/RBV with the lower ITT SVR), then, the model predicts all settings could achieve a 10% absolute reduction in chronic prevalence within 10 years. At 10 years, HCV chronic prevalence among PWID was projected to be 37–50% (95% interval) in Manchester, 22–35% in Bristol, 22–36% in East London, 23–34% in Nottingham, 14–24% in Plymouth, 9–18% in North Wales and 4–12% in Dundee.

**Figure 3:**
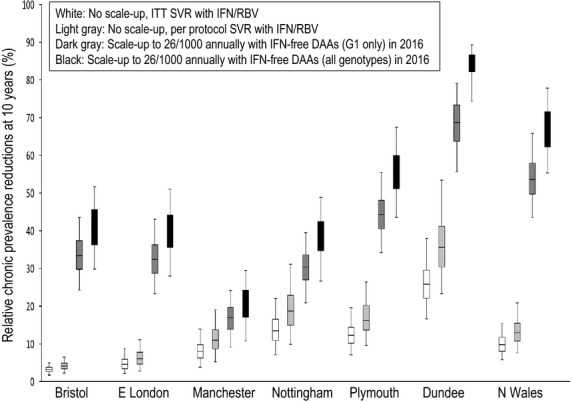
Relative HCV chronic prevalence reductions among PWID at 10 years assuming various treatment scenarios. Projections are shown for no treatment scale-up and using Peg-IFN/RBV ITT SVR rates from 2005 (white), no treatment scale-up and using Peg-IFN/RBV per protocol SVR rates from 2005 (light gray), ‘conservative DAA scale-up scenario’ with ITT SVR until 2015, treatment scale-up to 26/1000 PWID and IFN-free DAAs for genotype 1 patients only in 2016 (dark gray), ‘optimistic DAA scale-up scenario’ with per protocol SVR until 2015, treatment scale-up to 26/1000 PWID and IFN-free DAAs for all genotypes in 2016 (black). Boxes show the interquartile range, with whiskers indicating the 95% intervals.

Finally, if treatment rates are scaled up to 26 per 1000 PWID annually and IFN-free DAAs are available for all genotypes in early 2016 (Fig.2b), then, all settings could achieve a 15% absolute reduction in chronic prevalence within 10 years (95% interval predicting 14–20% absolute reduction in Bristol, 13–20% in East London, 7–16% in Manchester, 12–19% in Nottingham, 16–21% in Plymouth, 18–22% in Dundee and 18–22% in North Wales). Greater relative impact is seen in lower prevalence areas, with relative prevalence reductions of 42–63% (95% interval) in Plymouth, 70–86% in Dundee and 53–74% in North Wales (Fig.3).

#### ANCOVA analysis

The ANCOVA analysis (appendix Figure S1) indicates that the majority of variation in the impact projections is due to uncertainty in baseline treatment rate (contributing >35% of uncertainty in East London, Nottingham, Plymouth, Dundee and North Wales), injecting duration (contributing >25% uncertainty in Bristol, Manchester and Nottingham) and baseline chronic prevalence (contributing >20% uncertainty in Bristol, East London and Dundee). Less impact is achieved if the baseline chronic prevalence is towards the higher end of the estimates, if treatment rates are towards the lower end of the estimates and if injecting duration is at the shorter range of estimates.

## Discussion

We determined the current levels of HCV treatment for people who inject drugs (PWID) and SVR rates for Peg-IFN/RBV in a range of sites in the United Kingdom and showed that these were unlikely to achieve observable reductions in HCV prevalence over the next 10 years. However, with the introduction of IFN-free DAAs in 2016 and scale up of HCV treatment to rates achieved at two sites (∽26 per 1000 PWID or from 29 to 156 treatments annually in the sites), then all sites could achieve at least a 15% absolute reduction in HCV prevalence with relative reductions ranging from 12% to 86% after 10 years. Greater impact is achieved in sites with lower HCV prevalence, although we also show that uncertainty on key parts of the evidence (such as PWID prevalence and baseline treatment rates) leads to substantial variation in the projected impact.

We collected data on over 500 PWID – more than previous systematic reviews and other observational cohorts [18]. Our model projections, however, are subject to considerable uncertainty because of uncertainty around some key influential parameters.

First, the true duration of injecting drug use is difficult to estimate and remains uncertain [34]. However, we believe that duration is likely to be prolonged (with average duration >8–10 years) [30], therefore increasing the opportunity for HCV treatment to avert HCV infections [17]. Second, despite the largest public health surveillance programme in Europe, local estimates of HCV prevalence are uncertain and contribute to over 1/3 of the variability in model projections [35].

Third, we may have misclassified some PWID (as current injecting status was unknown for 6% of the sample, a proportion of people in OST may have a very low risk of relapse and a proportion of those that had last injected greater than 3 years also may relapse). However, this variability is dwarfed by the uncertainty in the denominator – the prevalence of PWID. We are very concerned over the credibility and reliability of estimates of PWID prevalence in general– which is a key input to the model projections – and an important source of caution for the projections [36]. Until more reliable and consistent estimates are generated, there will inevitably be uncertainty over the impact of treatment scale-up on HCV incidence and prevalence.

Fourth, we revealed uncertainty in the SVR with Peg-IFN/RBV – due in part to heterogeneity in local sites but primarily due to missing follow-up information. Additionally, PWID who have been previously treated may be subject to selection bias, and outcomes may not be representative if treatment were to be scaled up to the broader PWID population. However, it was strength that the reported SVR was consistent with published estimates from trials and other observational studies [18,19]. Although SVR of new DAAs in ‘real world’ remains unmeasured, we expect it to be greater than current treatments. Additionally, although we explore a scenario where IFN-free DAAs are only approved for genotype 1 patients, it is unclear whether they will immediately be funded and made available for all disease stages (such as mild fibrosis). Nevertheless, given the rapid developments in HCV therapy, an all-oral, pan-genotype, high-efficacy treatment for all stages is likely in the very near future [21–28].

Finally, we assumed no change in risk behaviour following treatment such that reinfection incidence equals primary incidence, despite evidence that reinfection rates among PWID are low [19]. If risk behaviour is reduced following treatment, then, our assumption provides conservative projections of impact. However, we again note that it is unclear whether sustained reductions in risk would be seen if treatment were scaled up to the broader PWID population.

Our study builds on previous work that has shown that scaling-up HCV treatment among injectors can lead to substantial reductions in HCV chronic prevalence among PWID and that HCV treatment is critical to primary prevention of HCV [11–16]. A recent modelling study estimated that scaling-up HCV DAA treatment in the general population could dramatically reduce the number of chronic infections in England [37], but did not include dynamic transmission of HCV among PWID, the risk of reinfection after treatment or the population prevention benefit of treatment of PWID. Therefore, in settings with ongoing HCV transmission, dynamic epidemic transmission models as used in this manuscript are required to generate robust estimates of the impact of treatment on the burden of chronic infection.

There are a growing number of studies that have shown that HCV treatment can be delivered to PWID with little loss in SVR compared to non or ex-PWID [18,19] and also that treatment can be expanded among PWID, especially if linked to OST [38–42]. Our model projections (based on real data) show that relatively high treatment rates can be achieved (at >20 per 1000 PWID) and could be sufficient to demonstrate ‘treatment as prevention’ in many settings. However, we show also a fourfold variation in HCV treatment rates of PWID across our sites in the United Kingdom- with the lowest rates at approximately 5 per 1000 PWID equivalent to rates in Vancouver and Melbourne [13]. Such variation represents the opportunities, but also the difficulties, for scaling-up HCV treatment. Some sites prioritized HCV treatment scale-up earlier than others and may have found it easier to establish effective services in the community, while others have encountered difficulties in scaling-up traditional HCV treatments among PWID – many of whom continue to experience chaotic lives and may not be in OST long enough to sustain HCV case finding and treatment [43–45].

Information on HCV treatment rates of PWID generally is lacking. One of the key challenges to the study was collecting information on PWID status, which was not always recorded on routine clinic databases and in several instances had to be collected from clinical notes [46]. However, it is important that routine clinical and surveillance data on HCV record information on OST and PWID status –so that policymakers and clinicians can monitor HCV treatment rates in order both to inform treatment target setting and to assess whether health inequalities are being reduced.

A key question is whether sufficient HCV treatment scale-up (to observe a reduction in HCV prevalence) can only be achieved through the introduction of new interferon-free DAAs. Despite promising treatment rates in some sites, we believe interferon-based therapy is unlikely to be a suitable agent to provide significant enhancement of treatment rates as it is unlikely to be acceptable to all patients or lend itself to extensive development of community-based treatment services. Introducing and scaling up HCV treatment with new IFN-free DAAs, however, as a public health ‘treatment as prevention’ intervention, are unlikely to be affordable at current protease inhibitor (PI) or DAA list prices, although full economic models comparing different treatment strategies have not yet been published. We have shown that optimizing OST and high-coverage NSP can reduce the number of HCV treatments required to reduce HCV prevalence [17]. We show here also that a similar intervention impact can be achieved by conserving new DAAs for genotype 1 only. We highlight the uncertainties surrounding two important parameters (PWID prevalence and local estimates of HCV prevalence in PWID) that need to be resolved. Finally, it is urgent that our model projections of the effectiveness and impact of HCV treatment as prevention are tested empirically.

## Abbreviations

CI: confidence intervals
DAA: direct acting antivirals
HCV: hepatitis C virus
ITT: intention to treat
NSP: needle and syringe programmes
OST: opiate substitution therapy
pegIFN: pegylated interferon
PWID: people who inject drugs
RBV: ribavirin
SVR: sustained viral response

## Supporting Information

Appendix: Service evaluation details

## References

[1] Shepard CW, Finelli L, Alter MJ (2005). Global epidemiology of hepatitis C virus infection. Lancet Infect Dis.

[2] Cooke GS, Lemoine M, Thursz M (2013). Viral hepatitis and the Global Burden of Disease: a need to regroup. J Viral Hepatitis.

[3] Ly KN, Xing J, Klevens RM, Jiles RB, Ward JW, Holmberg SD (2012). The increasing burden of mortality from viral hepatitis in the United States between 1999 and 2007. Ann Intern Med.

[4] Advisory Council on Misuse of Drugs (2009). The Primary Prevention of Hepatitis C Among Infecting Drug Users.

[5] Alter MJ, Moyer LA (1998). The importance of preventing hepatitis C virus infection among injection drug users in the United States. J Acquir Immune Defic Syndr Hum Retrovirol.

[6] De Angelis D, Sweeting M, Ades A, Hickman M, Hope V, Ramsay M (2009). An evidence synthesis approach to estimating Hepatitis C prevalence in England and Wales. Stat Methods Med Res.

[7] Harris RJ, Ramsay M, Hope VD (2012). Hepatitis C prevalence in England remains low and varies by ethnicity: an updated evidence synthesis. Eur J Public Health.

[8] Turner KM, Hutchinson S, Vickerman P (2011). The impact of needle and syringe provision and opiate substitution therapy on the incidence of hepatitis C virus in injecting drug users: pooling of UK evidence. Addiction.

[9] Van Den Berg C, Smit C, Van Brussel G, Coutinho R, Prins M (2007). Full participation in harm reduction programmes is associated with decreased risk for human immunodeficiency virus and hepatitis C virus: evidence from the Amsterdam Cohort Studies among drug users. Addiction.

[10] Vickerman P, Martin N, Turner K, Hickman M (2012). Can needle and syringe programmes and opiate substitution therapy achieve substantial reductions in hepatitis C virus prevalence? Model projections for different epidemic settings. Addiction.

[11] Martin NK, Vickerman P, Foster GR, Hutchinson SJ, Goldberg DJ, Hickman M (2011). Can antiviral therapy for hepatitis C reduce the prevalence of HCV among injecting drug user populations? A modeling analysis of its prevention utility. J Hepatol.

[12] Martin NK, Miners A, Vickerman P (2012). The cost-effectiveness of HCV antiviral treatment for injecting drug user populations. Hepatology.

[13] Martin NK, Vickerman P, Grebely J (2013). HCV treatment for prevention among people who inject drugs: modeling treatment scale-up in the age of direct-acting antivirals. Hepatology.

[14] Zeiler I, Langlands T, Murray JM, Ritter A (2010). Optimal targeting of Hepatitis C virus treatment among injecting drug users to those not enrolled in methadone maintenance programs. Drug Alcohol Depend.

[15] Durier N, Nguyen C, White LJ (2012). Treatment of hepatitis C as prevention: a modeling case study in Vietnam. PLoS ONE.

[16] Rolls D, Sacks-Davis R, Jenkinson R (2013). Hepatitis C transmission and treatment in contact networks of people who inject drugs. PLoS ONE.

[17] Martin NK, Hickman M, Hutchinson SJ, Goldberg DJ, Vickerman P (2013). Combination interventions to prevent HCV transmission among people who inject drugs: modeling the impact of antiviral treatment, needle and syringe programs, and opiate substitution therapy. Clin Infect Dis.

[18] Hellard M, Sacks-Davis R, Gold J (2009). Hepatitis C treatment for injection drug users: a review of the available evidence. Clin Infect Dis.

[19] Aspinall EJ, Corson S, Doyle JS (2013). Treatment of hepatitis C virus infection among people who are actively injecting drugs: a systematic review and meta-analysis. Clin Infect Dis.

[20] Harris R, Thomas B, Griffiths J (2014). Increased uptake and new therapies are needed to avert rising hepatitis C-related end stage liver disease in England: modelling the predicted impact of treatment under different scenario. J Hepatol.

[21] Jacobson IM, Gordon SC, Kowdley KV (2013). Sofosbuvir for hepatitis C genotype 2 or 3 in patients without treatment options. N Engl J Med.

[22] Lawitz E, Mangia A, Wyles D (2013). Sofosbuvir for previously untreated chronic hepatitis C infection. N Engl J Med.

[23] Lawitz E, Poordad FF, Pang PS (2014). Sofosbuvir and ledipasvir fixed-dose combination with and without ribavirin in treatment-naive and previously treated patients with genotype 1 hepatitis C virus infection (LONESTAR): an open-label, randomised, phase 2 trial. Lancet.

[24] Poordad F, Lawitz E, Kowdley KV (2013). Exploratory study of oral combination antiviral therapy for hepatitis C. N Engl J Med.

[25] Feld JJ, Kowdley KV, Coakley E (2014). Treatment of HCV with ABT-450/r–Ombitasvir and Dasabuvir with Ribavirin. N Engl J Med.

[26] Sulkowski MS, Gardiner DF, Rodriguez-Torres M (2014). Daclatasvir plus sofosbuvir for previously treated or untreated chronic HCV infection. N Engl J Med.

[27] Afdhal N, Zeuzem S, Kwo P (2014). Ledipasvir and Sofosbuvir for Untreated HCV Genotype 1 Infection. N Engl J Med.

[28] Everson G, Tran T, Towner W

[29] Health Protection Agency (2012). Hepatitis C in the UK 2012.

[30] Kimber J, Copeland L, Hickman M (2010). Survival and cessation in injecting drug users: prospective observational study of outcomes and effect of opiate substitution treatment. BMJ.

[31] Nosyk B, Anglin MD, Brecht ML, Lima VD, Hser YI (2013). Characterizing durations of heroin abstinence in the California Civil Addict Program: results from a 33-year observational cohort study. Am J Epidemiol.

[32] Booth JCL, O'Grady J, Neuberger J (2001). Clinical guidelines on the management of hepatitis C. Gut.

[33] Briggs A, Claxton K, Sculpher M (2006). Decision Modelling for Health Economic Evaluation.

[34] Sweeting MJ, De Angelis D, Ades AE, Hickman M (2008). Estimating the prevalence of ex-injecting drug use in the population. Stat Methods Med Res.

[35] Harris R, Hope V, Morongiu A, Hickman M, Ncube F, De Angeles D (2012). Spatial mapping of hepatitis C prevalence in recent injecting drug users in contact with services. Epidemiol Infect.

[36] Jones HE, Hickman M, Welton NJ, De Angelis D, Harris RJ, Ades AE (2014). Recapture or Precapture? Fallibility of Standard Capture-Recapture Methods in the Presence of Referrals Between Sources. Am J Epidemiol.

[37] Wedemeyer H, Duberg AS, Buti M (2014). Strategies to manage hepatitis C virus (HCV) disease burden. J Viral Hepatitis.

[38] Jafferbhoy H, Miller MH, Dunbar JK, Tait J, McLeod S, Dillon JF (2012). Intravenous drug use: not a barrier to achieving a sustained virological response in HCV infection. J Viral Hepat.

[39] Bruggmann P, Falcato L, Dober S (2008). Active intravenous drug use during chronic hepatitis C therapy does not reduce sustained virological response rates in adherent patients. J Viral Hepat.

[40] Witteck A, Schmid P, Hensel-Koch K, Thurnheer MC, Bruggmann P, Vernazza P (2011). Management of hepatitis C virus (HCV) infection in drug substitution programs. Swiss Med Wkly.

[41] Reimer J, Schulte B, Castells X (2005). Guidelines for the treatment of hepatitis C virus infection in injection drug users: status quo in the European Union countries. Clin Infect Dis.

[42] Schulte B, Schutt S, Brack J (2010). Successful treatment of chronic hepatitis C virus infection in severely opioid-dependent patients under heroin maintenance. Drug Alcohol Depend.

[43] Jack K, Willott S, Manners J, Varnam MA, Thomson BJ (2009). Clinical trial: a primary-care-based model for the delivery of anti-viral treatment to injecting drug users infected with hepatitis C. Aliment Pharmacol Ther.

[44] Tait JM, McIntyre PG, McLeod S, Nathwani D, Dillon JF (2010). The impact of a managed care network on attendance, follow-up and treatment at a hepatitis C specialist centre. J Viral Hepat.

[45] Irving WL, Smith S, Cater R (2006). Clinical pathways for patients with newly diagnosed hepatitis C - what actually happens. J Viral Hepat.

[46] Alavi M, Grebely J, Micallef M (2013). Assessment and treatment of hepatitis C virus infection among people who inject drugs in the opioid substitution setting: ETHOS study. Clin Infect Dis.

