# Supplementary material for: HCV treatment rates and sustained viral response among people who inject drugs in seven UK sites: real world results and modelling of treatment impact
Source: J Viral Hepat. 2014 Oct 7;22(4):399–408. doi: 10.1111/jvh.12338 (PMC4409099; doi:10.1111/jvh.12338)
Supplement: Supplementary file 1 — Appendix: Service evaluation details [file jvh0022-0399-sd1.docx]

**Service evaluation details**

Information on HCV prevalence was provided by public health surveillance (Unlinked Anonymous Monitoring (UAM) Survey of PWID in England and Wales([1](#_ENREF_1)), Needle Exchange Surveillance Initiative (NESI) in Scotland([2](#_ENREF_2)), and enhanced blood borne viral surveillance in Wales([3](#_ENREF_3))) and additional local studies([4-7](#_ENREF_4)). Information on PWID prevalence can vary because of different methodological approaches, even when based on the same data sources([8-10](#_ENREF_8)). For example, the number of PWID in England and Scotland vary two–fold from 120,000 to 200,000 and 15,000 to 30,000 respectively([11-15](#_ENREF_11)). We, therefore, adjusted national published estimates (in order to account for differences between these and other updated estimates (**Table S2**)), and calculated the HCV treatment rate per 1000 PWID for model projections. Here the denominator is the total number of PWID (not the estimated number with chronic HCV).

**Mathematical model**

We use a previously published dynamic, deterministic, compartmental model of HCV transmission and treatment among PWID([16](#_ENREF_16), [17](#_ENREF_17)). The model included compartments for uninfected PWID (*X(t)*), PWID chronically infected with HCV (*C(t)*), PWID on antiviral treatment (*T(t)*), and PWID who fail antiviral treatment (F*(t)*). We track changes in the populations over time, *t*. As the model is dynamic, the risk of infection or reinfection for a PWID is proportional to HCV chronic prevalence, which changes over time. We do not assume any risk difference after treatment; reinfection risk is equal to primary infection risk.

The full model equations are as follows


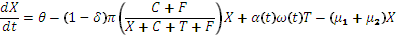


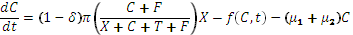


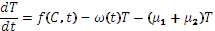


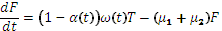


New injectors enter the susceptible PWID population at a rate (θ) individuals per year. Uninfected PWID can become infected with HCV at a rate proportional to the chronic prevalence of HCV and the infection rate, π. A proportion (δ, 26%([18](#_ENREF_18))) of individuals spontaneously clear their acute infection; the remainder (1- δ) progress to chronic infection (*C*), where we assume they are eligible for treatment. Here, f(*C*, *t*) is the number of chronically infected PWID initiated onto treatment per year. A fixed total number (Φ(t)) of PWID are initiated onto treatment per year in the population, with a treatment duration of 1/ω(t). If Φ(t) is greater than the number of eligible chronic infections, all eligible chronic infections are treated.


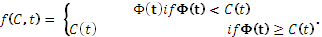


We note the treatment rate is denoted as annual numbers treated per total PWID population size (X+C+T+F). If treated, a proportion (α(t)) achieve SVR and return to the uninfected compartment. Those who do not attain SVR (1-α(t)) move to the treatment failure compartment. Due to the rapid reduction in viral loads during treatment([19](#_ENREF_19)), even for many who relapse, we assumed PWID are not infectious during treatment. We assumed treatment failures cannot be retreated. If retreatment were allowed, the impact projections would be slightly greater than our projections. PWID exit all compartments due to permanent cessation of drug use (μ_1_) or death due to drug or non-drug related causes (μ_2_). We neglect acute infection and immunity in the model as previous analyses have shown that their inclusion has minimal impact on model projections([17](#_ENREF_17), [20](#_ENREF_20)).

*Injecting duration*

Site-specific estimates of the average duration of injecting until long-term cessation are unavailable and difficult to obtain due to bias. Sweeting et al. ([12](#_ENREF_12)), combined UK population surveys and other information on PWID in order to adjust for sample biases, subsequently estimating 11 years from initiation to permanent cessation (95% confidence interval 6-20). Therefore we sample from a wide uncertainty interval for the uncertainty and sensitivity analyses. We use the same duration of injection across all the sites, sampling from a uniform distribution with mean of 11 years and a range of 6-16 years.

*IFN-free DAAs*

We assume IFN-free DAAs with a 90% SVR rate and 12 weeks treatment duration will be available in 2016 (for only genotype 1 patients, or all genotypes) in line with our previous modeling analyses([21](#_ENREF_21)) and clinical studies([22-29](#_ENREF_22)).

| **Parameter** | **Symbol** | **Units** | **Value [sampled range]** | **Ref-erence** |
| --- | --- | --- | --- | --- |
| Average proportion of infections that spontaneously clear | δ | - | 0.26 | ([18](#_ENREF_18)) |
| Average duration of injection until final cessation | 1/μ_1_ | Years | 11 [6-16, uniform dist.] | ([12](#_ENREF_12)) See text |
| Average death rate | μ_2_ | Per year | 0.01 [Poisson dist] |  |
| Average new injector rate | θ | Per 1000 PWID annually | Varied to fit a total population of 1000 PWID |  |
| Average infection rate per year | π | Per year | [0-0.95] Varied in each setting to fit to HCV chronic prevalence |  |
| SVR rate  pegIFN+RBV G1 ITT  pegIFN+RBV G2/3 ITT  pegIFN+RBV G1 per protocol  pegIFN+RBV G 2/3 per protocol  IFN-free DAAs | α* | - | 45% [33-57%, uniform dist]  61% [46-76%, uniform dist]  59% [46-71%, uniform dist]  82% [69-94%, uniform dist]  90% | ([22-29](#_ENREF_22)) |
| Duration of treatment  pegIFN+RBV G1 SVR  pegIFN+RBV G1 nonSVR  pegIFN+RBV G2/3  IFN-free DAAs | ω** | weeks | 48  12  24  12 | ([30](#_ENREF_30))  ([30](#_ENREF_30))  ([22-29](#_ENREF_22)) |
| HCV chronic prevalence among PWID  Bristol  East London  Manchester  Nottingham  Plymouth  Dundee & Tayside  Betsi Cadwaladr/North Wales |  | - | 43% [37-48%, uniform dist]  43% [37-48%, uniform dist]  52% [48-56%, uniform dist]  41% [37-44%, uniform dist]  33% [30-37%, uniform dist]  23% [20-27%, uniform dist]  30% [27-33%, uniform dist] | ([1-7](#_ENREF_1)) |
| Proportion genotype 1 | g_1_ | - | 46% | Weighted mean from this study |

**Table S1. Parameters used for the mathematical model simulations.** *Calculated using a weighted average of SVR proportions by genotype. **Calculated using a weighted average of treatment duration by SVR proportion and genotype. PWID: people who use drugs; SVR: sustained viral response; G1: genotype 1; G 2/3: genotypes 2 and 3; ITT: intention to treat; pegIFN+RBV: pegylated interferon and ribavirin; IFN-free DAA: interferon-free direct acting antiviral therapy; dist.: distribution

| **Parameter** | **Estimate or Range** | **Source** |
| --- | --- | --- |
| Number PWID  Bristol  East London  Manchester  Nottingham  Plymouth  Tayside/Dundee  North Wales | 3200-4400^^^  2400-6000^*^  2300-4000^*^  1300-2500^*^  1100-2000^*^  2000-3000^+^  1700-3400^~^ | *Estimated from Home Office estimates in England (Hay et al([31](#_ENREF_31), [32](#_ENREF_32))) adjusted by Harris et al([33](#_ENREF_33)).  ^local estimates (Hickman et al([34](#_ENREF_34)))  +ISD estimates in Scotland with adjustments by King et al([14](#_ENREF_14), [35](#_ENREF_35))  ^~^Health Protection Wales |
| Adult population age **15-59**  Bristol  East London  Manchester (city)  Nottingham  Plymouth  Tayside/Dundee  North Wales | 279,764  584,624  350,084  207,218  158.074  241,433  383,395 | ([36](#_ENREF_36)) |
| PWID prevalence  Bristol  East London  Manchester  Nottingham  Plymouth  Tayside/Dundee  North Wales | 1.14-1.57%  0.41-1.03%  0.66-1.14%  0.63-1.21%  0.7-1.27%  0.83-1.24%  0.44-0.89% | Calculated from above, uncertainty due to uncertainty in number of PWID |
| Number PWID treated per year  Bristol  East London  Manchester  Nottingham  Plymouth  Tayside/Dundee  North Wales | 18  25  63  32  17  34  18 | Data from service evaluation |
| Treatment rate per 1000 PWID in 2013  Bristol  East London  Manchester  Nottingham  Plymouth  Tayside/Dundee  North Wales | 4.1-5.6  4.2-10.4  15.8-27.4  12.8-24.6  8.5-15.5  11.3-17.0  5.3-10.6 | Calculated from above, uncertainty due to uncertainty in number of PWID |

**Table S2. Parameters used to estimate PWID treatment rates.**

(a) (b)


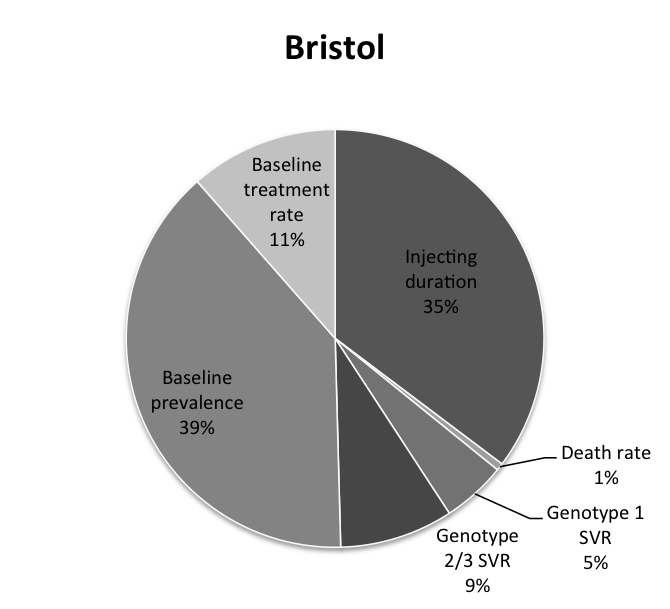

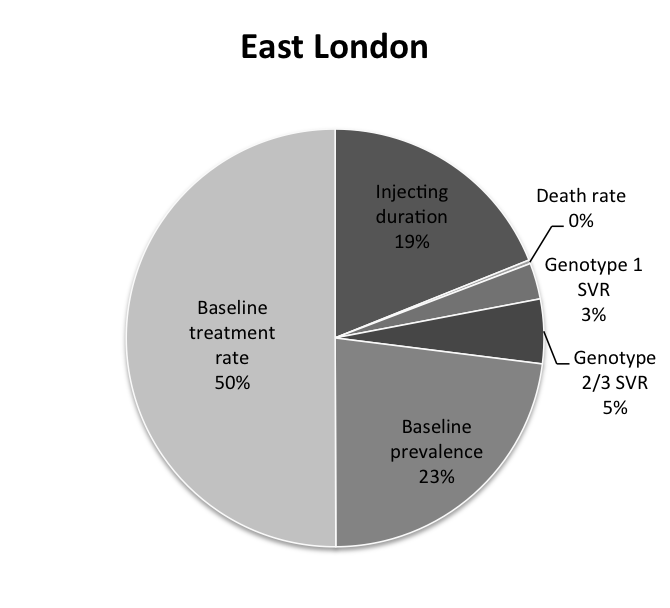


(c) (d)


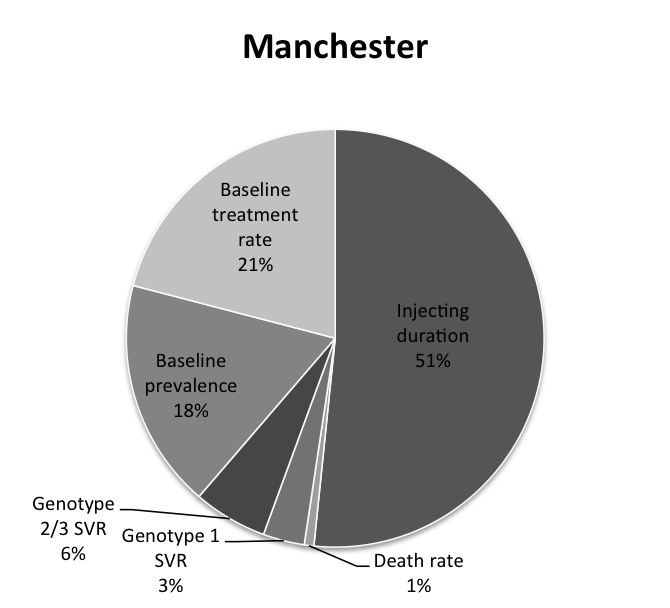

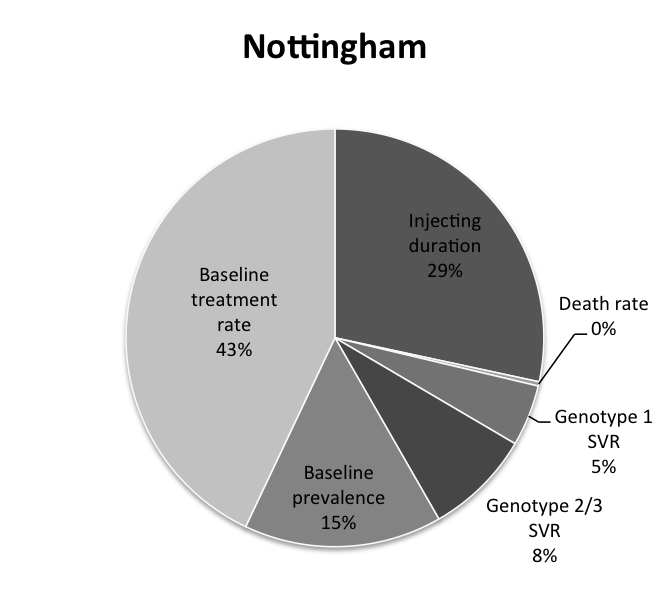


(e) (f)


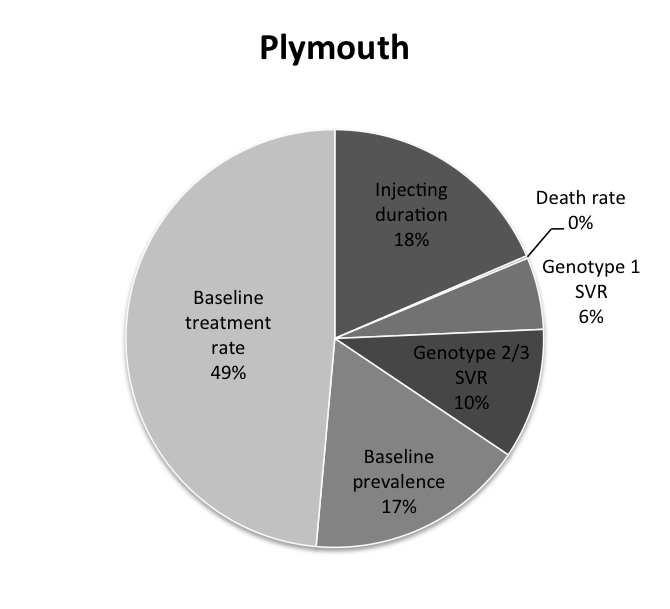

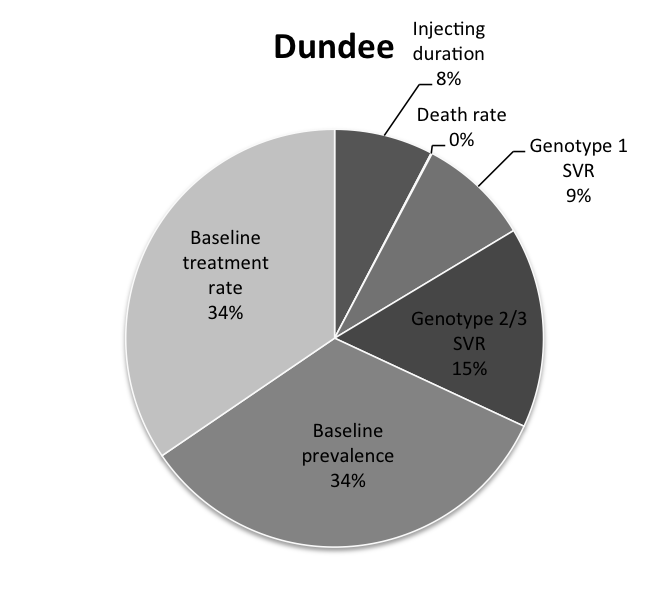


(g)


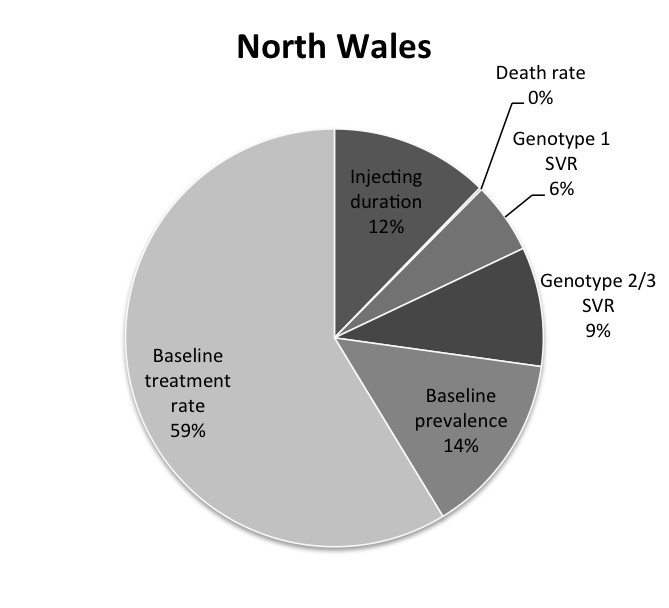


**Figure S1. Results of the ANCOVA analysis showing the proportion of the uncertainty in relative chronic prevalence decrease at 10 years resulting from uncertainty in each parameter.** Results shown using the ITT SVR scenario with pegIFN/RBV.

**References**

1. Health Protection Agency. Data tables of the Unlinked Anonymous Monitoring Survey of HIV and Hepatitis in Injecting Drug Users Surveillance Update: 2012. 2012.

2. University of the West of Scotland, Health Protection Scotland, and West of Scotland Specialist Virology Centre. The Needle Exchange Surveillance Initiative (NESI): Prevalence of HCV and injecting risk behaviours among injecting drug users attending injecting equipment provision services in Scotland, 2008/2009 & 2010. 2012.

3. Public Health Wales. Enhanced surveillance of blood borne viruses in drug users in Wales Annual report. 2012.

4. Harris RJ, Hope VD, Morongiu A, Hickman M, Ncube F, De AD. Spatial mapping of hepatitis C prevalence in recent injecting drug users in contact with services. Epidemiol Infect. 2011 8/30/2011:1-10.

5. Craine N, Walker AM, Williamson S, Brown A, Hope VD. Hepatitis B and hepatitis C seroprevalence and risk behaviour among community-recruited drug injectors in North West Wales. Commun Dis Public Health. 2004 9/2004;7(3):216-9.

6. Hickman M, Hope V, Brady T, Madden P, Jones S, Honor S, et al. Hepatitis C virus (HCV) prevalence, and injecting risk behaviour in multiple sites in England in 2004. J Viral Hepat. 2007 9/2007;14(9):645-52.

7. Hope VD, Hickman M, Ngui SL, Jones S, Telfer M, Bizzarri M, et al. Measuring the incidence, prevalence and genetic relatedness of hepatitis C infections among a community recruited sample of injecting drug users, using dried blood spots. J Viral Hepat. 2011 4/27/2011;18:262-70.

8. Hickman M, Hope V, Coleman B, Parry J, Telfer M, Twigger J, et al. Assessing IDU prevalence and health consequences (HCV, overdose and drug-related mortality) in a primary care trust: implications for public health action. JPublic Health (Oxf). 2009 9/2009;31(3):374-82.

9. Hickman M, De AD, Jones H, Harris R, Welton N, Ades AE. Multiple parameter evidence synthesis-a potential solution for when information on drug use and harm is in conflict. Addiction. 2013 4/22/2013.

10. Jones HE, Hickman M, Welton NJ, De Angelis D, Harris RJ, Ades AE. Recapture or Precapture? Fallibility of Standard Capture-Recapture Methods in the Presence of Referrals Between Sources. Am J Epidemiol. 2014 Apr 11. PubMed PMID: 24727806.

11. Sweeting MJ, D. DA, Hickman M, Ades AE. Estimating hepatitis C prevalence in England and Wales by synthesizing evidence from multiple data sources. Assessing data conflict and model fit. Biostatistics. 2008 10/2008;9(4):715-34.

12. Sweeting MJ, De Angelis D, Ades AE, Hickman M. Estimating the prevalence of ex-injecting drug use in the population. Stats Meth Med Res. 2009 August 1, 2009;18(4):381-95.

13. Hay G, Gannon M, MacDougall J, Eastwood C, Williams K, Millar T. Capture--recapture and anchored prevalence estimation of injecting drug users in England: national and regional estimates. Stat Methods Med Res. 2009 Aug;18(4):323-39. PubMed PMID: 19036919.

14. King R, Bird S, Hay G, Hutchinson S. Estimating current injectors in Scotland and their drug-related death rate by sex, region and age-group via Bayesian capture-recapture methods. Statistical Methods in Medical Research. 2009 November 26, 2008:0962280208094701.

15. Overstall AM, King R, Bird SM, Hutchinson SJ, Hay G. Incomplete contingency tables with censored cells with application to estimating the number of people who inject drugs in Scotland. Stat Med. 2014 Apr 30;33(9):1564-79. PubMed PMID: 24293386.

16. Martin NK, Vickerman P, Foster GR, Hutchinson SJ, Goldberg DJ, Hickman M. Can antiviral therapy for hepatitis C reduce the prevalence of HCV among injecting drug user populations? A modelling analysis of its prevention utility. Journal of Hepatology 2011;54:1137-44.

17. Martin NK, Vickerman P, Hickman M. Mathematical modelling of Hepatitis C Treatment for Injecting Drug Users. Journal of Theoretical Biology. 2011;274:58-66.

18. Micallef JM, Kaldor JM, Dore GJ. Spontaneous viral clearance following hepatitis C infection: a systematic review of longitudinal studies. J Viral Hepat. 2006;13:34-41.

19. Dahari H, Ribeiro RM, Perelson AS. Triphasic decline of hepatitis C virus RNA during antiviral therapy. Hepatology. 2007;46(1):16-21.

20. Vickerman P, Martin N, Turner K, Hickman M. Can needle and syringe programmes and opiate substitution therapy achieve substantial reductions in HCV prevalence? Model projections for different epidemic settings. Addiction. 2012;107:1984-95.

21. Martin N, Vickerman P, Grebely J, Hellard M, Hutchinson S, Lima V, et al. HCV treatment for prevention among people who inject drugs: modeling treatment scale-up in the age of direct-acting antivirals. Hepatology. 2013;58(5):1598-609.

22. Jacobson IM, Gordon SC, Kowdley KV, Yoshida EM, Rodriguez-Torres M, Sulkowski MS, et al. Sofosbuvir for Hepatitis C Genotype 2 or 3 in Patients without Treatment Options. New England Journal of Medicine. 2013;368(20):1867-77. PubMed PMID: 23607593.

23. Lawitz E, Mangia A, Wyles D, Rodriguez-Torres M, Hassanein T, Gordon SC, et al. Sofosbuvir for Previously Untreated Chronic Hepatitis C Infection. New England Journal of Medicine. 2013;368(20):1878-87. PubMed PMID: 23607594.

24. Lawitz E, Poordad FF, Pang PS, Hyland RH, Ding X, Mo H, et al. Sofosbuvir and ledipasvir fixed-dose combination with and without ribavirin in treatment-naive and previously treated patients with genotype 1 hepatitis C virus infection (LONESTAR): an open-label, randomised, phase 2 trial. The Lancet. (0).

25. Poordad F, Lawitz E, Kowdley KV, Cohen DE, Podsadecki T, Siggelkow S, et al. Exploratory Study of Oral Combination Antiviral Therapy for Hepatitis C. N Engl J Med. 2013;368(1):45-53.

26. Feld JJ, Kowdley KV, Coakley E, Sigal S, Nelson DR, Crawford D, et al. Treatment of HCV with ABT-450/r–Ombitasvir and Dasabuvir with Ribavirin. New England Journal of Medicine. 2014;370(17):1594-603. PubMed PMID: 24720703.

27. Sulkowski MS, Gardiner DF, Rodriguez-Torres M, Reddy KR, Hassanein T, Jacobson I, et al. Daclatasvir plus Sofosbuvir for Previously Treated or Untreated Chronic HCV Infection. New England Journal of Medicine. 2014;370(3):211-21. PubMed PMID: 24428467.

28. Afdhal N, Zeuzem S, Kwo P, Chojkier M, Gitlin N, Puoti M, et al. Ledipasvir and Sofosbuvir for Untreated HCV Genotype 1 Infection. New England Journal of Medicine. 2014;DOI: 10.1056/NEJMoa1402454. PubMed PMID: 24725239.

29. Everson G, Tran T, Towner W, et al. Safety and efficacy of treatment with the interferon-free, ribavirin-free combination of sofosbuvir + GS-5816 for 12 weeks in treatment naive patients with genotype 1-6 HCV infection. 49th European Association for the Study of the Liver International Liver Congress (EASL 2014) 2014;Abstract O111.

30. NICE. Peginterferon alfa and ribavirin for the treatment of mild chronic hepatitis C. Technol Appraisal Guidance 106. 2006.

31. Hay G, Gannon M, MacDougall J, Eastwood C, Williams K, Millar T. Capture-recapture and anchored preavlence estimation of injecting drug users in England: national and regional estimates. Statistical Methods in Medical Research. 2009;18(4):323-39.

32. Hay G, Gannon M, macDougal J, Millar T, Eastwood C, Williams K, et al. Estimates of the prevalence of opiate use and/or crack cocaine use (2006/07) South West Region. 2008.

33. Harris R, Hope V, Morongiu A, Hickman M, Ncube F, De Angeles D. Spatial mapping of hepatitis C prevalence in recent injecting drug users in contact with services. Epidemiology & Infection. 2012;140(06):1054-63.

34. Hickman M, Hope V, Coleman B, Parry J, Telfer M, Twigger J, et al. Assessing IDU prevalence and health consequences (HCV, overdose and drug-related mortality) in a primary care trust: implications for public health action. J Public Health. 2009 July 16, 2009:1-9.

35. King R, Bird SM, Overstall A, Hay G, Hutchinson SJ. Injecting drug users in Scotland, 2006: Listing, number, demography, and opiate-related death-rates. Addiction Research & Theory. 2013;21(3):235-46.

36. Office for National Statistics. Population Estimates for UK, England and Wales, Scotland and Northern Ireland. 2012.
